# High prevalence of malignancy in HIV-positive patients with mediastinal lymphadenopathy: A study in the era of antiretroviral therapy

**DOI:** 10.1111/resp.12241

**Published:** 2014-01-29

**Authors:** JOANA ALÇADA, MAGALI N TAYLOR, PENNY J SHAW, SAM M JANES, NEAL NAVANI, ROBERT F MILLER

**Affiliations:** 1 Department of Thoracic Medicine, University College London HospitalsLondon, UK; 3 Lungs for Living Research Centre, UCL RespiratoryLondon, UK; 4 Research Department of Infection and Population Health, University College LondonLondon, UK; 2 Department of Radiology, University College London HospitalsLondon, UK; 5 Department of Clinical Research, Faculty of Infections and Tropical Diseases, London School of Hygiene and Tropical MedicineLondon, UK

**Keywords:** antiretroviral agent, computed tomography scan, human immunodeficiency virus, lymph node, mediastinum

## Abstract

**Background and objective:**

Mediastinal lymphadenopathy (MLN) in human immunodeficiency virus (HIV) infection has a wide spectrum of aetiologies with different prognoses and treatments. The decision to pursue a histopathological diagnosis represents a clinical challenge as patients present with non-specific symptoms. This study aimed to determine the aetiology and predictive factors of MLN in a cohort of HIV-infected patients in the combination antiretroviral therapy (cART) era.

**Methods:**

Single-centre retrospective cohort study of 217 consecutive HIV-infected patients who underwent computed tomography (CT) of the chest between January 2004 and December 2009. Fifty-two patients were identified to have MLN (>10 mm in short axis). CT images were re-reviewed by an independent radiologist blinded to the clinical information. Final diagnoses of MLN were obtained from clinical records. Multivariate analysis was performed to identify predictors of aetiology of MLN.

**Results:**

Seventeen patients (33%) had a diagnosis of malignancy. Consolidation on CT was associated with a reduced likelihood of malignancy odds ratio (OR) 0.03 (95% confidence interval 0.002–0.422), and larger lymph nodes were associated with an increase in the odds of malignancy (OR 2.89; 95% confidence interval 1.24–6.71). CD4 count was found not to be a predictor of aetiology of MLN.

**Conclusions:**

In the era of combination cART, opportunistic infections and malignancy remain to be the frequent causes of MLN in HIV-positive patients, but the prevalence of non-HIV related malignancy has increased compared with previous studies. Although certain findings are predictors of non-malignant disease, pathological diagnosis of MLN in HIV-positive patients should be pursued whenever possible.

**SUMMARY AT A GLANCE**

We evaluated radiological, laboratory and clinical data of HIV patients with MLN in the era of combination ART. Opportunistic infections and malignancy remain frequent causes of MLN in HIV-positive patients, but the prevalence of non-HIV-related malignancy has increased compared with previous studies, emphasizing the need for histological diagnosis.

## Introduction

Combination antiretroviral therapy (cART) has changed the landscape of human immunodeficiency virus (HIV) infection, with significant declines in associated morbidity and mortality. Prior to the advent of cART, pulmonary disorders were among the most common complications of HIV infection. They were predominantly infectious diseases and were linked to unfavourable outcomes.^1^–^3^ With the introduction of cART, the epidemiology of pulmonary complications emerging in successfully treated HIV-infected patients has shifted. The spectrum includes fewer infections, fewer acquired immune deficiency syndrome-defining cancers and an increased frequency of chronic obstructive pulmonary disease^4^,^5^ and non-acquired immune deficiency syndrome-defining cancers, particularly lung cancer.^6^,^7^

Mediastinal lymphadenopathy (MLN) has been previously reported to occur in 35–40% of patients infected with HIV.^8^,^9^ The broad range of aetiologies and the non-specific clinical presentation represent a diagnostic challenge.^8^,^10^

The utility of chest computed tomography (CT) for assessing pulmonary disease in HIV infection has been widely explored and is a key initial radiological investigation.^9^ It allows characterization of non-specific radiographic patterns and detection of occult lung disease as well as assessment of the mediastinum and airways.^11^ However, the role of chest CT in the aetiological diagnosis of MLN in patients with HIV infection is not well characterized. In addition, previously published series were conducted prior to the era of cART.^8^,^9^ Although nodal biopsy is ultimately the gold standard for clarifying the aetiology of MLN,^12^–^14^ it is important to identify whether clinical, laboratory or radiological predictors can be used to guide or obviate the need for invasive sampling. Such variables may, in fact, be useful in predicting a specific diagnosis and thus help developing a tailored management strategy in this group of patients.

To our knowledge, no study to date has focused on the mode of diagnosis of MLN in HIV-infected patients in the era of cART. In this study, we aimed to identify the causes of MLN in HIV-infected patients presenting to a specialist unit and to characterize their mode of diagnosis and follow-up. We also aimed to identify clinical and radiological predictors of MLN aetiology in this patient group.

## Methods

### Participants, setting and data collection

**Figure 1:**
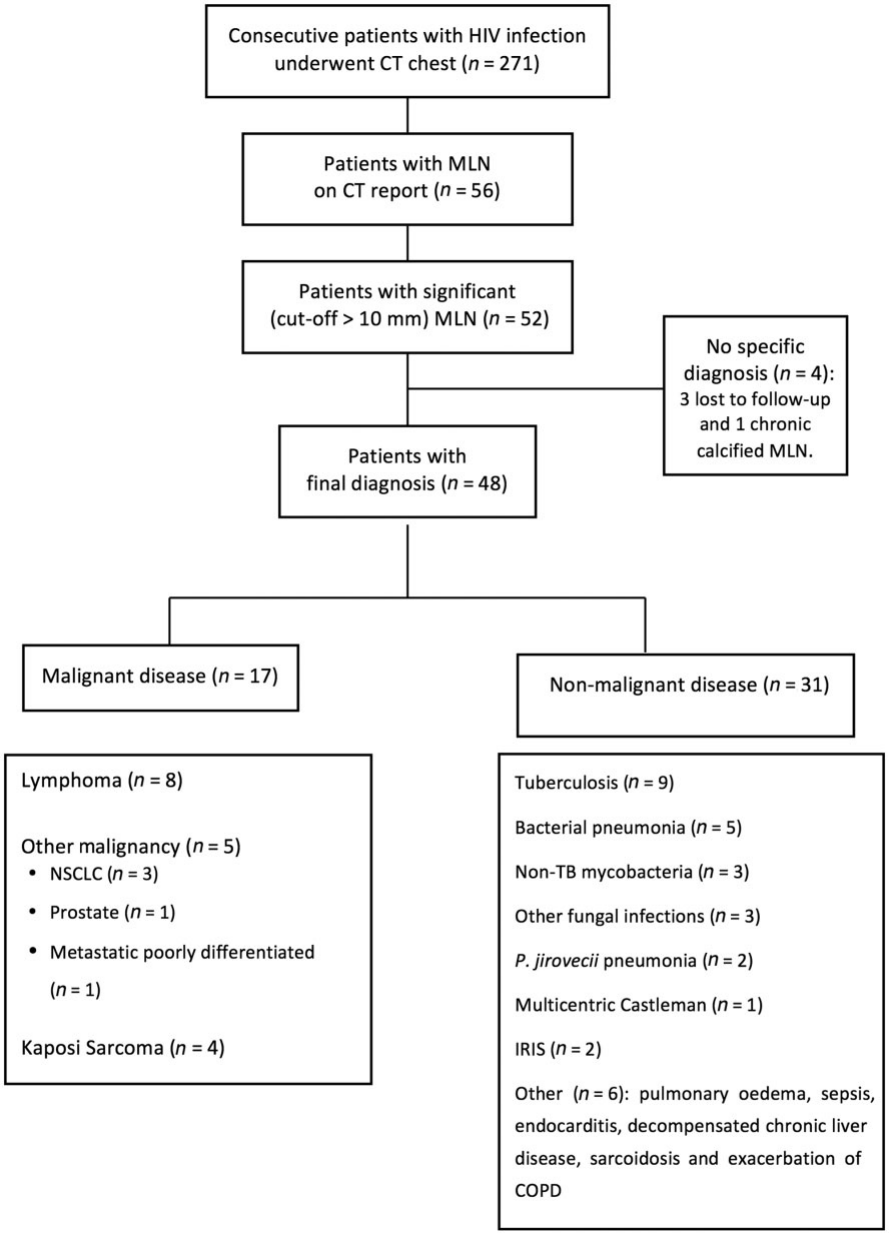
Flowchart of human immunodeficiency virus (HIV)-positive patients undergoing computed tomography (CT) chest diagnosed with intrathoracic lymphadenopathy. COPD, chronic obstructive pulmonary disease; IRIS, immune reconstitution inflammation syndrome; MLN, mediastinal lymphadenopathy; NSCLC, non-small-cell lung cancer; TB, tuberculosis.

We conducted a retrospective analytical cohort study of HIV-positive patients who underwent CT scanning of the chest at University College London Hospitals between 1 January 2004 and 31 December 2009 for investigation of respiratory symptoms. For patients with more than one scan, only their initial CT scan was included for analysis. Data were collected from January 2010 through January 2012. A total of 271 consecutive patients were identified. All patients were known to be HIV-1 antibody positive at the time of scanning. Clinical indications for CT were collected for all 271 patients (Fig. 1).

CT scan of the thorax was performed using an electron beam scanner (Siemens SOMATOM Sensation Multislice CT scanner, Siemens Healthcare AG, Erlangen, Germany). CT images were reviewed in the standard axial plane, with coronal reconstructions, on both mediastinal (soft tissue) and lung window settings. CT slice thickness varied between 1 and 3 mm. The scanning protocols adopted were: standard arterial phase chest CT (36-s post-intravenous contrast), high-resolution CT and CT pulmonary angiogram depending on the clinical indication at the time.

All original CT reports were reviewed by the same observer (J.A.), and MLN was initially identified in 56 patients. These CT images were then evaluated independently by one thoracic radiologist (M.T.) who was blinded to all clinical data, except for HIV status. The conventional CT cut-off for significant intrathoracic lymphadenopathy of 10 mm in short-axis^15^ was employed.

The radiological features were recorded using the Fleischner Society Glossary of Terms for Thoracic Imaging.^16^ Location and size of MLN, presence of necrosis and of extrathoracic lymphadenopathy were recorded. The lung parenchyma was evaluated for presence of consolidation, ground-glass opacification, cavity or cyst formation, lung nodules and airways disease (indicated by emphysema, bronchial wall thickening or bronchiectasis).

Baseline demographic, laboratory and clinical data were collected from electronic medical records and included age, sex, HIV risk factors, ethnicity, most recent CD4 cell count and duration of receipt of cART. All 52 patients were followed up for at least 12 months after the initial assessment.

### Diagnostic criteria

Final diagnoses of MLN were based on positive pathology or microbiology and clinical and radiological follow-up of at least 6 months duration. Definitions for particular conditions can be found in Appendix S1 in the online supporting information.

### Statistical analysis

For descriptive analysis, 52 patients with HIV infection and MLN on CT scan of the thorax were included. For univariate and multivariate analysis of CT findings, CD4 cell count and duration of cART as predictors of specific aetiology, we restricted our analysis to patients with a definitive final diagnosis (*n* = 48). Due to the sample size and to allow correct modelling, we restricted our analysis to groups of aetiologies accounting for at least a third of patients.

CD4 cell counts were converted into categorical variables and stratified into three groups >500, 200–500, and <200 cells/mm^3^ in accordance with the 2008 Center for Disease Control and Prevention Revised Classification System for HIV infection.^17^ Times between CT and start of cART were converted into dichotomous variables (before and after CT).

For univariate analysis, odds ratios (OR) and 95% confidence intervals were calculated to assess the risk for having cancer (lymphoma, lung cancer, other malignancy). A two-sided *P*-value ≤0.05 was used to define statistical significance.

For multivariate analysis, we calculated adjusted OR for radiological and laboratory findings associated with malignancy by using logistic regression with backward elimination (likelihood ratios). Variables were chosen based on statistical significance (*P* < 0.1) in univariate analysis or previously published evidence.^8^,^9^

Statistical analysis was performed using the software SPSS version 18 (IBM Corporation, Armonk, NY, USA) and Stata Version 10 (Statcorp, Houston, TX, USA).

The observational nature of the study meant that ethical approval was not required, as determined by the United Kingdom National Research Ethics Service. The design, analysis and report of this study conform to the Standard of Reporting Diagnostic Accuracy statement.^18^

## Results

Two hundred seventy-one consecutive patients underwent chest CT in the study period. All patients were HIV-1 antibody-positive. Significant (>10 mm) intrathoracic lymphadenopathy was present in 52 patients (19%). A specific diagnosis was made in 48 cases (92%). Of the four patients without a diagnosis, three were lost to follow-up, and one patient had persistent calcified MLN attributed to previous mycobacterial infection.

### Clinical indication for CT

Abnormal chest X-ray (21.8%), weight loss plus night sweats and/or fever (10.3%), and shortness of breath (5.9%) were the three commonest clinical indications for CT (Table S1 in the online supporting information for full details).

### Demographic, clinical and pathological characteristics

The median age (interquartile range) of the population with intrathoracic lymphadenopathy was 40 (35–44) years, and their demographic characteristics are summarized in Table 1.

**Table 1:** Demographic characteristics of the study population

Variable	Number (%)
Age, median years (IQR)	40 (35–44)
Sex	
Male	46 (88.5)
Female	6 (11.5)
Ethnicity	
White	32 (61.5)
African Caribbean	12 (23.1)
Asian	2 (3.8)
Other	6 (11.5)
HIV risk factor†	
MSM	28 (53.8)
Heterosexual	20 (38.5)
Bisexual	1 (1.9)
Vertical transmission	1 (1.9)
Intravenous drug use	5 (9.6)

† One heterosexual patient was infected through vertical transmission; one MSM patient and one heterosexual patient were intravenous drug users.

IQR, interquartile range; MSM, men who have sex with men.

Twenty-four (46%) patients were receiving cART, 19 were cART-naïve, and cART status was unclear for nine (17%) patients. There were no relevant differences in demographic characteristics between the cART/cART-naïve patients. The majority (61%) had started cART at least 1 year prior to undergoing CT thorax. Seventeen (90%) of the 19 cART-naïve patients had started cART following CT.

Twenty-seven patients had a CD4 lymphocyte count below 250 cells × 10^6^/L at the time of CT (see Table 2).

**Table 2:** Aetiologies of MLN and CD4 count at time of diagnosis

	CD4 lymphocyte count (cells × 10^6^/L)†				
Diagnosis	<200	200–500	>500	Unknown	Total *n* (%)
Tuberculosis	7	1	1	0	9 (17)
Non-tuberculous mycobacteria	1	1	1	0	3 (6)
PCP	1	1	0	0	2 (4)
Bacterial pneumonia	1	3	1	0	5 (10)
Other fungal infections§	0	2	1	0	3 (6)
Lymphoma	2	3	1	2	8 (13.3)
Other malignancy‡	1	1	2	1	5 (10)
Kaposi sarcoma	1	1	2	0	4 (8)
Castleman's disease	0	1	0	0	1 (2)
IRIS	2	0	0	0	2 (4)
Other¶	2	3	1	0	6 (11)
Unknown				4	4 (8)
Total	18	17	10	7	52

† CD4 groups according to CDC surveillance case definition for HIV.^17^

‡ Three patients with non-small cell lung cancer (one poorly differentiated non-small cell lung cancer, one anaplastic large cell carcinoma and one squamous cell lung cancer), one patient with prostate cancer and one patient with metastatic poorly differentiated carcinoma.

§ One patient with *Aspergillosis*, one patient with *Cryptococcus*, one patient with *Candida*.

¶ One patient with pulmonary oedema, one patient with sepsis, one patient with endocarditis, one patient with decompensated chronic liver disease, one patient with sarcoidosis and one patient with infective exacerbation of chronic obstructive pulmonary disease.

CDC, Center for Disease Control and Prevention; HIV, human immunodeficiency virus; IRIS, immune reconstitution inflammatory syndrome; MLN, mediastinal lymphadenopathy; PCP, *Pneumocystis jirovecii* pneumonia.

### Spectrum of disease

The aetiologies of MLN are summarized in Table 2. Tuberculosis was the single most common diagnosis accounting for nine (17%) cases. In total, 17 patients (33%) had a diagnosis of malignancy with lymphoma accounting for eight (15%) cases. Two cases of immune reconstitution inflammation syndrome (IRIS) were identified.

In patients on cART, lymphoma (17%) and bacterial pneumonia (13%) were the commonest diagnoses with tuberculosis accounting for 8% of cases only. In cART-naïve patients, tuberculosis (26%) was the commonest diagnosis, with lymphoma and bacterial pneumonia accounting for 11% of cases each.

### Mode of diagnosis

Final diagnoses were established by histopathology in 24 cases (50%), histopathology with microbiology in five cases (10%), microbiology in seven (15%), and clinical and/or radiological follow-up in 11 patients (23%). Flexible bronchoscopy was done in nine cases (17%).

Histopathological sampling was performed using ultrasound-guided aspiration of peripheral lymph nodes (ultrasound-guided fine needle aspiration) (*n* = 19), mediastinoscopy (*n* = 3) or endobronchial ultrasound-guided transbronchial needle aspiration (*n* = 3).

### CT findings

Using the Mountain–Dresler lymph node map,^19^ the most frequently involved mediastinal node stations were 2, 4, 7 and 8 (see Table 3). Lymph node size varied from 1 to 7 cm with a median size (interquartile range) of 1.55 cm (1.28–2.55).

**Table 3:** Distribution of enlarged mediastinal lymph nodes by station^19^

LN station	Number of patients (*n*)
1	19
2	33
3	27
4	30
5	19
6	15
7	30
8	30
9	6
10	28
11	8

LN, lymph node.

The radiological features observed for each diagnosis are shown in Table S2 in the online supporting information. Extrathoracic lymphadenopathy, lung nodules and consolidation were the three most common radiological findings and were observed in 58% (*n* = 30), 54% (*n* = 28) and 37% (*n* = 19) of patients, respectively. Extrathoracic lymphadenopathy was a feature in six cases (75%) of lymphoma and all cases of Kaposi's sarcoma.

### Clinical and radiographic predictors

In the logistic regression model for predicting a diagnosis of malignancy, the following covariates were included: age, ethnicity, HIV risk factor, absolute CD4 count, parenchymal CT findings, presence of extrathoracic lymphadenopathy, lymph node maximum size and presence of necrotic lymph nodes.

In the univariate analysis, increased size of lymph nodes appeared to be associated with an increased risk of malignancy, whereas lung consolidation on CT appeared to be associated with non-malignant disease.

The multivariate analysis confirmed that the presence of consolidation on CT scan significantly reduced the OR of malignancy in the MLN (OR 0.03, 95% confidence interval 0.002–0.422; *P* = 0.009). It also confirmed that larger lymph nodes were more likely to be malignant, with an increase of 1 cm in short axis resulting in a 2.89 increase in the odds of malignancy (95% confidence interval 1.24–6.71; *P* = 0.014). Age, ethnicity, HIV risk factor, absolute CD4 count, extrathoracic lymphadenopathy and presence of necrotic lymph nodes were not associated with malignancy (Table 4).

**Table 4:** Univariate and multivariate analyses of factors predictive of malignancy in HIV-positive patients with MLN

Covariate	Unadjusted OR for malignancy	Univariate, *P*-value	Adjusted OR for malignancy (95% CI)	Multivariate, *P*-value
Age	1.022 (0.957–1.091)	0.522	—	—
Ethnicity	0.939 (0.665–1.327)	0.722	—	—
Sexual Orientation	1.564 (0.683–3.582)	0.290	—	—
Absolute CD4 count	1.533 (0.638–3.679)	0.339	—	—
LN necrosis	1.038 (0.264–4.089)	0.957	—	—
Lung parenchymal cavity/cyst formation	0.211 (0.024–1.845)	0.160	—	—
Extrathoracic LN	1.267 (0.539–2.982)	0.587	1.809 (0.530–6.163)	0.344
LN maximum size (cm)	1.452 (0.927–2.274)	0.103	2.890 (1.245–6.709)	**0.014**
Lung consolidation	0.141 (0.028–0.712)	0.018	0.031 (0.002–0.422)	**0.009**

—, not calculated as univariate *P*-value >0.1; CI, confidence interval; HIV, human immunodeficiency virus; LN, lymph node; MLN, mediastinal lymphadenopathy; OR, odds ratio.

## Discussion

This is the first study to assess the aetiology and mode of diagnosis of HIV-infected patients with MLN in the era of combination ART. Important findings have been made that impact diagnosis and management.

Significant MLN was a common finding (19%) in HIV-infected patients undergoing chest CT for assessment of respiratory symptoms. Malignancy was a frequent diagnosis, accounting for a third of cases (33%) with non-HIV-related malignancies representing a significant proportion of neoplasms (29%). IRIS was identified for the first time as an aetiology of MLN. Radiographic findings that may be useful predictors of the aetiology of MLN were identified. Finally, MLN sampling in this population is often necessary and can be successfully and safely accomplished by either endoscopic ultrasound-fine needle aspiration or endobronchial ultrasound-guided transbronchial needle aspiration.

Two other previously published studies addressed the significance of MLN in HIV-infected patients;^8^,^10^ however, both were undertaken in the pre-ART era. The frequency of MLN in our study (20%) is comparable with the series by Jasmer *et al*. (35%).^8^ In both series tuberculosis, bacterial pneumonia and lymphoma were the three most common diagnosis, whereas mycobacterial disease and lymphoma accounted for the majority of cases in the Fishman and Sagar series.^10^ However, some differences can be observed in the spectrum of disease. Compared with Jasmer *et al*., our series shows an important decline in the diagnosis of bacterial pneumonia (21% vs 10%) and a marked increase in the number of non-HIV related malignancies (3% vs 11%).

In our series, we also describe two cases of IRIS, which were not identified by Jasmer *et al*. To our knowledge, this is the first time IRIS is reported as aetiology of MLN in HIV-infected patients. Although it has been associated with a range of opportunistic infections, mycobacteria are the infections most commonly implicated in IRIS.^20^ In our series, one of the IRIS cases was associated with *Mycobacterium avium* complex infection. The patient had a baseline CD4 count of 70 cells × 10^6^/L and presented with fever and lymphadenitis within 1 month of starting combination ART. This is in line with the typical presentation of *Mycobacterium avium* complex IRIS.^21^ The other patient with IRIS had been diagnosed with cryptococcal meningitis the year before, and cART was started at that stage.

The observed differences can have several explanations. First, and perhaps more importantly, the study periods were different with the Jasmer *et al*. series reporting findings when cART was not available. In fact, when stratified by cART status, the spectrum of disease of the cART-naïve group was similar to that observed by Jasmer *et al*. Our findings are also consistent with the results from several recent studies^5^,^22^–^24^ that have shown a change in the overall spectrum of pulmonary disease in patients with HIV infection with the use of cART. In fact, data from two major longitudinal studies^22^,^24^ have demonstrated a reduction in the rates of bacterial pneumonia and the emergence of IRIS associated with antiretroviral treatment. Other studies have demonstrated that the proportion of non-HIV-related cancers has increased significantly with the use of cART.^25^,^26^ Differences between study populations, particularly ethnicity (our population was predominantly White (61.5%), whereas in Jasmer *et al*., White and African American ethnic groups accounted for 43% and 41% of cases) and HIV risk factors (intravenous drug use 9.6% vs 30%), may also explain the variation in the results.

Previous studies established necrotic lymph nodes for mycobacterial disease, airways disease for bacterial pneumonia and absence of pulmonary nodules for lymphoma as radiological predictors for those aetiologies.^8^ In our series, multivariate analysis demonstrated that the presence of consolidation (OR 0.03, *P* = 0.009) is an independent predictor for having a diagnosis of non-malignant disease, whereas larger lymph nodes increase the likelihood (OR 2.89, *P* = 0.014) of malignancy. The results demonstrate that the high frequency of MLN and the wide spectrum of disease pose important diagnostic challenges. The high prevalence of malignancy in this setting also emphasizes the need for a pathological diagnosis where possible. In our study, 68% of histological samples were obtained by ultrasound-guided aspiration of peripheral lymph nodes, underlining the importance of a thorough physical exam. For MLN sampling, mediastinoscopy was performed as frequently as endobronchial ultrasound-guided transbronchial needle aspiration. This may owe to the limited availability of endobronchial ultrasound-guided transbronchial needle aspiration throughout the first years covered by our study. Recent studies have demonstrated that endobronchial ultrasound-guided transbronchial needle aspiration can obviate the need for mediastinoscopy in patients with MLN.^14^,^27^

Our study has a number of limitations. Data were obtained retrospectively from electronic records review. However, we attempted to enhance the validity of the results by independent blinded review of the chest CT scans with prospective recording of findings. Due to the retrospective design, there were no standardized criteria for having a CT scan of the chest and so it is difficult to exclude some selection bias. However, our study is consistent with previous reports in regards to incidence of MLN and main diagnoses.^8^,^9^ Finally, this is a single-centre study with a relatively small sample size, and therefore, the results may not be generalizable, as significant discrepancies in demographics, access to care, mode of transmission, and availability of cART are common among the HIV-infected population.

In the era of cART, opportunistic infections remain frequent causes of MLN in HIV-positive patients, but malignancy now may have a higher prevalence. Although certain findings are predictors of non-malignant disease, pathological diagnosis of MLN in HIV-positive patients should be pursued whenever possible.

## Abbreviations

cART: combination antiretroviral therapy
CT: computed tomography
HIV: human immunodeficiency virus
IRIS: immune reconstitution inflammatory syndrome
MLN: mediastinal lymphadenopathy
OR: odds ratio.

## Supporting information

Table S1 Clinical indications for CT scan of the chest in the study population.

Table S2 Radiological features by diagnosis.

Appendix S1 Diagnostic criteria for different aetiologies of mediastinal lymphadenopathy in HIV-infected patients.
