# Supplementary material for: High prevalence of malignancy in HIV-positive patients with mediastinal lymphadenopathy: A study in the era of antiretroviral therapy
Source: Respirology. 2014 Jan 29;19(3):339–45. doi: 10.1111/resp.12241 (PMC4016741; doi:10.1111/resp.12241)
Supplement: Table S1 — Clinical indications for CT scan of the chest in the study population. [file resp0019-0339-sd1.doc]

**Supporting Information**

**HIGH PREVALENCE OF MALIGNANCY in HIV positive patients with Mediastinal Lymphadenopathy: a study in the Era of Antiretroviral Therapy**

Joana Alçada ^1^, Magali N. Taylor^3^, Penny J. Shaw^3^, Sam M. Janes^1,2^, Neal Navani^1,2*^ and Robert F. Miller^4,5*^

1. Department of Thoracic Medicine, University College London Hospitals, London, UK
2. Lungs for Living Research Centre, UCL Respiratory, University College London
3. Department of Radiology, University College London Hospitals, London, UK
4. Research Department of Infection and Population Health, University College London, London, UK
5. Department of Clinical Research, Faculty of Infections and Tropical Diseases, London School of Hygiene and Tropical Medicine, London, UK

*Joint senior authors

**Diagnostic criteria for different aetiologies of mediastinal lymphadenopathy in HIV infected patients**

Tuberculosis was defined by typical clinical and radiographic presentation, with identification of *Mycobacterium tuberculosis* by direct microscopic examination of sputum or bronchoalveolar lavage (BAL) fluid or by culture of relevant specimens (sputum, BAL fluid, tissue biopsy or needle aspiration).^28^

*Mycobacterium avium complex* (MAC) infection was defined by identification of MAC from multiple respiratory specimens, body fluid, abscess fluid or tissue.^29^ ^30^

*Pneumocystis jirovecii* pneumonia (PCP) was defined by typical clinical and radiographic presentation, with demonstration of the cystic form of *P. jirovecii* in BAL fluid by Grocott methenamine silver staining and response to anti-Pneumocystis therapy.^31^

Bacterial pneumonia was defined by the acute onset of respiratory symptoms, focal or diffuse radiographic abnormalities, identification of a specific bacterial pathogen in BAL fluid and/or blood, and clinical and radiological response to antibiotics in conventional doses.^32^

Pulmonary Kaposi sarcoma was defined by direct visualization during bronchoscopy of typically red or violaceous, flat or raised discrete plaques, combined with identification of skin lesions.^33^

Diagnoses of lymphoma and lung cancer were defined by histopathological examination from either bronchoscopic, EBUS-TBNA, transthoracic needle, or surgical biopsy specimens.^12^ ^34^

Multicentric Castleman disease (MCD) was defined according to the French Agence Nationale de Recherche sur le SIDA) 117 CastlemaB trial group criteria: fever, CRP >20 mg/L in the absence of any other cause and three or more of peripheral lymphadenopathy, splenomegaly, jaundice, ascites, cough, autoimmune haemolytic anaemia and nasal obstruction.^35^ ^36^

Immune Reconstitution Inflammatory Syndrome (IRIS) was defined by presence of symptoms of infection or inﬂammatory disease, occurring after initiation, re-initiation, or change to a more effective combination antiretroviral therapy with associated decrease in viral load and/or increase in CD4 cell count, with those symptoms not explainable by a newly acquired infection or inﬂammatory condition or worsening of known infections or medication reaction.^20^ ^21^

Diagnoses of *Cryptococcus neoformans* were defined by isolation of the organism from sputum or BAL fluid.^37^ *Aspergillus* infection was defined by fungal culture or examination of pathologic specimens. ^38^

**Table S1 – Clinical indications for CT scan of the chest in the study population**

| **Indication for CT** | **N (%)** |
| --- | --- |
| Abnormal CXR | 59 (21.8%) |
| Weight Loss/Night Sweats ±fever | 28 (10.3%) |
| Shortness of Breath | 16 (5.9%) |
| Pyrexia of Unknown Origin | 15 (5.5%) |
| Follow-up | 14 (5.2%) |
| Cough | 13 (4.8%) |
| History of Cancer | 12 (4.4%) |
| LN | 9 (3.3%) |
| Pleuritic pain/chest pain | 6 (2.2%) |
| History of Tuberculosis | 6 (2.2%) |
| Poor clinical response to treatment | 3 (1.1%) |
| Haemoptysis | 2 (0.7%) |
| Information on HIV status only | 53 (19.6%) |
| Other | 35 (12.9%) |

**Table S2 - Radiological features by diagnosis**

|  | **Total** | Necrotic LN  n (%) | Extra-thoracic LN  n (%) | Pleural effusions  n (%) | Lung Nodules  n (%) | Consolidation  n (%) | Ground-glass opacification  n (%) | Cavity or cyst formation  n (%) | Airways disease  n (%) |
| --- | --- | --- | --- | --- | --- | --- | --- | --- | --- |
| TB | 9 | 5 (56) | 4 (44) | 1 (11) | 7 (78) | 5 (56) | 3 (33) | 3 (33) | 7 (78) |
| Kaposi Sarcoma | 4 | 0 | 4 (100) | 0 | 1 (25) | 0 | 0 | 0 | 0 |
| Atypical Mycobacteria | 3 | 1 (33) | 2 (67) | 0 | 1 (33) | 1 (33) | 1 (33) | 0 | 3 (100) |
| Other Fungal | 3 | 1 (33) | 0 | 1 (33) | 2 (67) | 2 (67) | 1 (33) | 0 | 1 (33) |
| Bacterial pneumonia | 5 | 0 | 3 (60) | 1 (20) | 1 (20) | 4 (80) | 3 (60) | 1 (20) | 2 (40) |
| Lymphoma | 8 | 2 (25) | 6 (75) | 0 | 2 (25) | 2 (25) | 0 | 0 | 4 (50) |
| IRIS | 2 | 1 (50) | 1 (50) | 0 | 1 (50) | 0 | 0 | 0 | 0 |
| Sarcoid | 1 | 0 | 0 | 0 | 1 (100) | 0 | 0 | 0 | 1 (100) |
| Unknown | 4 | 0 | 1 (25) | 1 (25) | 2 (50) | 1 (25) | 0 | 3 (75) | 1 (25) |
| Other | **5** | 0 | 4 (80) | 3 (60) | 3 (60) | 3 (60) | 1 (20) | 0 | 2 (40) |
| MCD | **1** | 0 | 1 (100) | 0 | 1 (100) | 0 | 1 (100) | 0 | 1 (100) |
| PCP | **2** | 0 | 1 (50) | 0 | 1 (50) | 1 (50) | 1 (50) | 1 (50) | 2 (100) |
| Other malignancy | **5** | 2 (40) | 3 (60) | 0 | 5 (100) | 0 | 0 | 1 (20) | 3 (60) |
| **Total** | **52** | **12 (23)** | **30 (58)** | **7 (13)** | **28 (54)** | **19 (37)** | **11 (21)** | **9 (17)** | **27 (52)** |

LN – lymph nodes, TB – Tuberculosis, IRIS - Immune Reconstitution Inflammatory Syndrome, MCD - Multicentric Castleman's Disease, PCP - Pneumocystis jirovecii pneumonia

| **REFERENCES** |
| --- |

1. Wallace JM, Hansen NI, Lavange L, Glassroth J, Browdy BL, Rosen MJ, Kvale PA, Mangura BT, Reichman LB, Hopewell PC. Respiratory disease trends in the Pulmonary Complications of HIV Infection Study cohort. Pulmonary Complications of HIV Infection Study Group. *Am. J. Respir. Crit. Care Med.* 1997;155(1):72–80.

2. Rosen MJ, Clayton K, Schneider RF, Fulkerson W, Rao AV, Stansell J, Kvale PA, Glassroth J, Reichman LB, Wallace JM, Hopewell PC. Intensive care of patients with HIV infection: utilization, critical illnesses, and outcomes. Pulmonary Complications of HIV Infection Study Group. *Am. J. Respir. Crit. Care Med.* 1997;155(1):67–71.

3. Miller R. HIV series HIV-associated respiratory diseases. *Lancet*. 1996;348:307–312.

4. Crothers K, Butt A A, Gibert CL, Rodriguez-Barradas MC, Crystal S, Justice AC. Increased COPD among HIV-positive compared to HIV-negative veterans. *Chest*. 2006;130(5):1326–33.

5. Crothers K, Huang L, Goulet JL, Goetz MB, Brown ST, Rodriguez-Barradas MC, Oursler KK, Rimland D, Gibert CL, Butt AA, Justice AC. HIV infection and risk for incident pulmonary diseases in the combination antiretroviral therapy era. *Am. J. Respir. Crit. Care Med.* 2011;183(3):388–95.

6. Shiels MS, Pfeiffer RM, Gail MH, Hall HI, Li J, Chaturvedi AK, Bhatia K, Uldrick TS, Yarchoan R, Goedert JJ, Engels EA. Cancer burden in the HIV-infected population in the United States. *J. Natl. Cancer Inst.* 2011;103(9):753–62.

7. Frisch M, Biggar RJ, Engels EA, Goedert JJ. Association of cancer with AIDS-related immunosuppression in adults. *JAMA J. Am. Med. Assoc.* 2001;285(13):1736–45.

8. Jasmer RM, Gotway MB, Creasman JM, Webb WR, Edinburgh KJ, Huang L. Clinical and radiographic predictors of the etiology of computed tomography-diagnosed intrathoracic lymphadenopathy in HIV-infected patients. *J. Acquir. Immune Defic. Syndr.* 2002;31(3):291–8.

9. Hartman TE, Primack SL, Müller NL, Staples CA. Diagnosis of thoracic complications in AIDS: accuracy of CT. *Am. J. Roentgenol.* 1994;162(3):547–53.

10. Fishman JE, Sagar M. Thoracic lymphadenopathy in HIV patients: spectrum of disease and differential diagnosis. *AIDS Patient Care STDS*. 1999;13(11):645–9.

11. Kang EY, Staples CA, McGuinness G, Primack SL, Müller NL. Detection and differential diagnosis of pulmonary infections and tumors in patients with AIDS: value of chest radiography versus CT. *Am. J. Roentgenol.* 1996;166(1):15–9.

12. De Leyn P, Lardinois D, Van Schil PE, Rami-Porta R, Passlick B, Zielinski M, Waller DA., Lerut T, Weder W. ESTS guidelines for preoperative lymph node staging for non-small cell lung cancer. *Eur. J. Cardio-Thoracic Surg.* 2007;32(1):1–8.

13. Lowe SM, Kocjan GI, Edwards SG, Miller RF. Diagnostic yield of fine-needle aspiration cytology in HIV-infected patients with lymphadenopathy in the era of highly active antiretroviral therapy. *Int. J. STD AIDS*. 2008;19(8):553–6.

14. Navani N, Molyneaux PL, Breen RA, Connell DW, Jepson A, Nankivell M, Brown JM, Morris-Jones S, Ng B, Wickremasinghe M, Lalvani A, Rintoul RC, Santis G, Kon OM, Janes SM. Utility of endobronchial ultrasound-guided transbronchial needle aspiration in patients with tuberculous intrathoracic lymphadenopathy: a multicentre study. *Thorax*. 2011;66(10):889–93.

15. Glazer GM, Gross BH, Francis IA, Bookstein FL, Orringer B. Number and Size According to American Thoracic Society Mapping Normal Nodes: Mediastinal Lymph. *Am. J. Roengtology*. 1985;(144):261–265.

16. Hansell DM, Bankier AA, MacMahon H, McLoud TC, Müller NL, Remy J. Fleischner Society: glossary of terms for thoracic imaging. *Radiology*. 2008;246(3):697–722.

17. Schneider E, Whitmore S, Glynn KM, Dominguez K, Mitsch A, McKenna MT. Revised surveillance case definitions for HIV infection among adults, adolescents, and children aged <18 months and for HIV infection and AIDS among children aged 18 months to <13 years. *MMWR Recomm. reports*. 2008;57(RR-10):1–12.

18. Bossuyt PM, Reitsma JB, Bruns DE, Gatsonis CA, Glasziou PP, Irwig LM, Lijmer JG, Moher D, Rennie D, de Vet HCW. Towards complete and accurate reporting of studies of diagnostic accuracy: the STARD initiative. *BMJ*. 2003;326(7379):41–4.

19. Mountain CF, Dresler CM. Regional lymph node classification for lung cancer staging. *Chest*. 1997;111(6):1718–23.

20. Lawn SD, Bekker L-G, Miller RF. Immune reconstitution disease associated with mycobacterial infections in HIV-infected individuals receiving antiretrovirals. *Lancet Infect. Dis.* 2005;5(6):361–73.

21. French MA, Price P, Stone SF. Immune restoration disease after antiretroviral therapy. *AIDS*. 2004;18(12):1615–1627.

22. Grubb JR, Moorman AC, Baker RK, Masur H. The changing spectrum of pulmonary disease in patients with HIV infection on antiretroviral therapy. *AIDS*. 2006;20(8):1095–107.

23. Hull MW, Phillips P, Montaner JSG. Changing global epidemiology of pulmonary manifestations of HIV/AIDS. *Chest*. 2008;134(6):1287–98.

24. Crothers K, Thompson BW, Burkhardt K, Morris A, Flores SC, Diaz PT, Chaisson RE, Kirk GD, Rom WN, Huang L. HIV-associated lung infections and complications in the era of combination antiretroviral therapy. *Proc. Am. Thorac. Soc.* 2011;8(3):275–81.

25. Bonnet F, Burty C, Lewden C, Costagliola D, May T, Bouteloup V, Rosenthal E, Jougla E, Cacoub P, Salmon D, Chêne G, Morlat P. Changes in Cancer Mortality among HIV‐Infected Patients: The Mortalité 2005 Survey. *Clin. Infect. Dis.* 2009;48(5):633–639.

26. Cheung MC, Pantanowitz L, Dezube BJ. AIDS-related malignancies: emerging challenges in the era of highly active antiretroviral therapy. *Oncologist*. 2005;10(6):412–26.

27. Navani N, Lawrence DR, Kolvekar S, Hayward M, McAsey D, Kocjan G, Falzon M, Capitanio A, Shaw P, Morris S, Omar RZ, Janes SM. Endobronchial ultrasound-guided transbronchial needle aspiration prevents mediastinoscopies in the diagnosis of isolated mediastinal lymphadenopathy: a prospective trial. *Am. J. Respir. Crit. Care Med.* 2012;186(3):255–60.

28. National Institute for Health and Clinical Excellence. Tuberculosis Clinical diagnosis and management of tuberculosis, and measures for its prevention and control. *NICE*. 2006;CG 33 (March).

29. Hammoud ZT, Anderson RC, Meyers BF, Guthrie TJ, Roper CL, Cooper JD PG. Diagnosis and treatment of disease caused by nontuberculous mycobacteria - Official statement of the American Thoracic Society. *Am. J. Respir. Crit. Care Med.* 1997;156(2 Pt 2):S1–25.

30. Griffith DE, Aksamit T, Brown-Elliott BA, Catanzaro A, Daley C, Gordin F, Holland SM, Horsburgh R, Huitt G, Iademarco MF, Iseman M, Olivier K, Ruoss S, Von Reyn CF, Wallace RJ, Winthrop K. An official ATS/IDSA statement: diagnosis, treatment, and prevention of nontuberculous mycobacterial diseases. *Am. J. Respir. Crit. Care Med.* 2007;175(4):367–416.

31. Miller RF, Millar AB, Weller I V, Semple SJ. Empirical treatment without bronchoscopy for Pneumocystis carinii pneumonia in the acquired immunodeficiency syndrome. *Thorax*. 1989;44(7):559–64.

32. Hirschtick RE, Glassroth J, Jordan MC, Wilcosky TC, Wallace JM, Kvale PA, Markowitz N, Rosen MJ, Mangura BT, Hopewell PC. Bacterial pneumonia in persons infected with the human immunodeficiency virus. Pulmonary Complications of HIV Infection Study Group. *N. Engl. J. Med.* 1995;333(13):845–51.

33. Miller RF, Tomlinson MC, Cottrill CP, Donald JJ, Spittle MF, Semple SJ. Bronchopulmonary Kaposi’s sarcoma in patients with AIDS. *Thorax*. 1992;47(9):721–725.

34. Eisner MD, Stulbarg MS, Kaplan LD, Herndier B. The Pulmonary Manifestations of AIDS-Related Non-Hodgkin’s Lymphoma. *Chest*. 1996;110(3):729–736.

35. Sayer R, Paul J, Tuke P, Hargreaves S, Noursadeghi M, Tedder R, Grant P, Edwards S, Miller R. Can plasma HHV8 viral load be used to differentiate multicentric Castleman disease from Kaposi sarcoma? *Int. J. STD AIDS*. 2011;22(10):585–589.

36. Gérard L, Bérezné A, Galicier L, Meignin V, Obadia M, De Castro N, Jacomet C, Verdon R, Madelaine-Chambrin I, Boulanger E, Chevret S, Agbalika F, Oksenhendler E. Prospective study of rituximab in chemotherapy-dependent human immunodeficiency virus associated multicentric Castleman’s disease: ANRS 117 CastlemaB Trial. *J. Cinical Oncol.* 2007;25(22):3350–6.

37. Cameron ML, Bartlett JA, Gallis HA, Waskin HA. Manifestations of Pulmonary Cryptococcusis in Patients with Acquired Immunodeficiency Syndrome. *Clin. Infect. Dis.* 1990;13(1):64–67.

38. Addrizzo-Harris DJ, Harkin TJ, McGuinness G, Naidich DP, Rom WN. Pulmonary Aspergilloma and AIDS: A Comparison of HIV-infected and HIV-Negative Individuals. *Chest*. 1997;111(3):612–618.
